# Supplementary material for: Neuroendocrine Response and State Anxiety Due to Psychosocial Stress Decrease after a Training with Subject’s Own (but Not Another) Virtual Body: An RCT Study
Source: Int J Environ Res Public Health. 2022 May 23;19(10):6340. doi: 10.3390/ijerph19106340 (PMC9140346; doi:10.3390/ijerph19106340)
Supplement: Supplementary file 1 [file ijerph-19-06340-s001.zip › ijerph-1692398-supplementary.pdf]

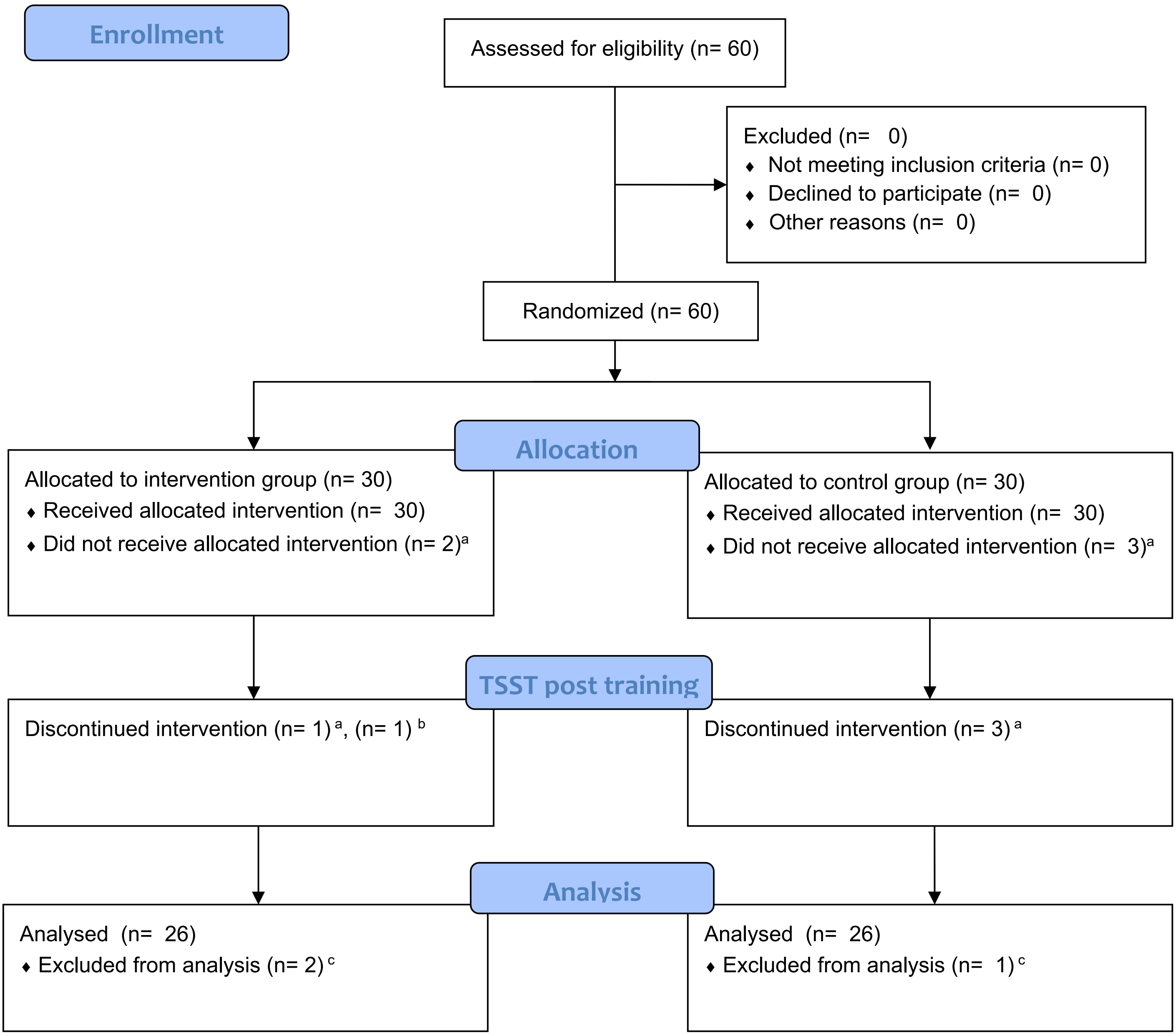

[a] subjects attended the first session but did not attend the second one (without giving any reason). therefore they did not receive the allocated intervention.

[b] subject discontinued the intervention because of motion sickness.

[c] females who were in the luteal menstrual phase during the first session but were in a different menstrual phase in the second did not meet inclusion criteria, therefore their data were excluded from analysis.

Figure S1. CONSORT 2010 Flow Diagram.

Table S1. CONSORT 2010 checklist of information to include when reporting a randomised trial.

Section/Topic	Item No.	Checklist Item	Reported on Page No.
**Title and abstract**			
	1a	Identification as a randomised trial in the title	1
	1b	Structured summary of trial design, methods, results, and conclusions (for specific guidance see CONSORT for abstracts)	1
**Introduction**			
Background and objectives	2a	Scientific background and explanation of rationale	1–2
	2b	Specific objectives or hypotheses	2
**Methods**			
Trial design	3a	Description of trial design (such as parallel, factorial) including allocation ratio	3
	3b	Important changes to methods after trial commencement (such as eligibility criteria), with reasons	5
Participants	4a	Eligibility criteria for participants	2–3
	4b	Settings and locations where the data were collected	2,3
Interventions	5	The interventions for each group with sufficient details to allow replication, including how and when they were actually administered	6–7
Outcomes	6a	Completely defined pre-specified primary and secondary outcome measures, including how and when they were assessed	6–8

	6b	Any changes to trial outcomes after the trial commenced, with reasons	not applicable
Sample size	7a	How sample size was determined	3
	7b	When applicable, explanation of any interim analyses and stopping guidelines	not applicable
Randomisation:			
Sequence generation	8a	Method used to generate the random allocation sequence	3
	8b	Type of randomisation; details of any restriction (such as blocking and block size)	3
Allocation concealment mechanism	9	Mechanism used to implement the random allocation sequence (such as sequentially numbered containers), describing any steps taken to conceal the sequence until interventions were assigned	3
Implementation	10	Who generated the random allocation sequence, who enrolled participants, and who assigned participants to interventions	3
Blinding	11a	If done, who was blinded after assignment to interventions (for example, participants, care providers, those assessing outcomes) and how	3
	11b	If relevant, description of the similarity of interventions	6–7
Statistical methods	12a	Statistical methods used to compare groups for primary and secondary outcomes	9–10
	12b	Methods for additional analyses, such as subgroup analyses and adjusted analyses	9–10
**Results**			

Participant flow (a diagram is strongly recommended)	13a	For each group, the numbers of participants who were randomly assigned, received intended treatment, and were analysed for the primary outcome	11–15, Supplementary Figure S1
	13b	For each group, losses and exclusions after randomisation, together with reasons	3, Supplementary Figure S1
Recruitment	14a	Dates defining the periods of recruitment and follow-up	2–3
	14b	Why the trial ended or was stopped	not applicable
Baseline data	15	A table showing baseline demographic and clinical characteristics for each group	Supplementary Table S2
Numbers analysed	16	For each group, number of participants (denominator) included in each analysis and whether the analysis was by original assigned groups	Supplementary Figure S1
Outcomes and estimation	17a	For each primary and secondary outcome, results for each group, and the estimated effect size and its precision (such as 95% confidence interval)	11–15
	17b	For binary outcomes, presentation of both absolute and relative effect sizes is recommended	not applicable
Ancillary analyses	18	Results of any other analyses performed, including subgroup analyses and adjusted analyses, distinguishing pre-specified from exploratory	12–15
Harms	19	All important harms or unintended effects in each group (for specific guidance see CONSORT for harms)	7

Discussion

Limitations	20	Trial limitations, addressing sources of potential bias, imprecision, and, if relevant, multiplicity of analyses	18
Generalisability	21	Generalisability (external validity, applicability) of the trial findings	16–17
Interpretation	22	Interpretation consistent with results, balancing benefits and harms, and considering other relevant evidence	16–18
**Other information**			
Registration	23	Registration number and name of trial registry	2
Protocol	24	Where the full trial protocol can be accessed, if available	not available
Funding	25	Sources of funding and other support (such as supply of drugs), role of funders	19

Table S2. Descriptive statistic of the sample. The first column summarizes all demographic details and the results of the questionnaires administered at the beginning of the RCT. Results are displayed as average (SE) or descriptively, when appropriate. None of them resulted to be significantly different between groups.

	**1PP**	**3PP**	**all**
N	26	26	52
gender	f = 12; m = 14	f = 12; m = 14	f = 24; m = 28
age	21.84 (0.28)	22.19 (0.31)	22.01 (0.24)
Body Mass Index	21.88 (0.46)	21.40 (0.60)	21.64 (0.37)
years of education	15.93 (0.33)	16.19 (0.28)	16.05 (0.21)
smoke habitudes (N, cigarettes per day)	1, 1–10; 1, 11–24	1, 1–10	2, 1–10; 1, 11–24
handedness	24 right-handed; 1 ambidextrous; 1 left-handed	23 right-handed; 2 ambidextrous; 1 left-handed	47 right-handed; 3 ambidextrous; 2 left-handed
hours of sleep before session 1	6.84 (0.21)	6.84 (0.22)	6.84 (0.15)
hours of sleep before session 2	7.01 (0.22)	6.76 (0.31)	6.89 (0.18)
HADS anxiety	4.53 (0.67)	4.65 (0.65)	4.59 (0.46)
HADS depression	5.50 (0.50)	5.46 (0.64)	5.48 (0.40)
PSS	24.61 (0.49)	24.61 (0.69)	24.61 (0.42)
STAI-T	40.11 (1.36)	42.42 (1.78)	41.26 (1.12)

IPAQ-kcal	2324.19 (534.24)	1858.19 (291.29)	2091.19 (303.01)
IPAQ score	2 low; 19 moderate; 5 high	1 low; 21 moderate; 4 high	3 low; 40 moderate; 9 high

Table S3. Results of Spearman's correlations between online (columns) and offline (rows) questionnaires' results. For each comparison, r and p values are displayed. * highlights significant correlations.

	**s1**	**s2**	**s3**	**s4**
**s5**	0.15	−0.19	0.23	0.04
	0.24	0.36	0.09	0.76
**s6**	0.39	−0.29	0.23	0.15
	0.01 *	0.03 *	0.06	0.51
**s7**	0.37	−0.24	0.32	−0.16
	0.01 *	0.14	0.04 *	0.41
**s8**	0.45	−0.49	0.44	0.04
	<0.01 *	<0.01 *	<0.01 *	0.48
**s9**	0.42	−0.41	0.46	0.02
	0.01 *	<0.01 *	<0.01 *	0.87
**s10**	−0.56	0.43	−0.56	0.12
	0.01 *	0.01 *	0.01 *	0.53
**s11**	0.07	−0.17	0.35	−0.01
	0.37	0.23	0.01 *	0.94
**s12**	0.02	−0.14	0.11	0.03
	0.61	0.41	0.48	0.48
**s13**	0.06	−0.05	0.30	−0.24
	0.79	0.92	0.04 *	0.03 *
**s14**	−0.45	0.23	−0.19	0.11
	<0.01 *	0.10	0.25	0.20
**s15**	−0.43	0.27	−0.30	0.24
	<0.01 *	0.01 *	0.04 *	0.42

Table S4. Results of Spearman's correlations between HR during the IVRt (column) and during the TSS for all time points (rows). For each comparison, r and p values are displayed. * highlights significant correlations.

	**HR during IVRt**
HR dt_1	−0.53<br><0.01 *
HR dt_2	−0.51<br><0.01 *
HR dt_3	−0.437<br><0.01 *
HR dt_4	−0.519<br><0.01 *
